# Long-term complications and implant survival rates after cranioplastic surgery: a single-center study of 392 patients

**DOI:** 10.1007/s10143-020-01374-4

**Published:** 2020-08-26

**Authors:** Henrik Giese, Jennifer Meyer, Andreas Unterberg, Christopher Beynon

**Affiliations:** Department of Neurosurgery, University of Heidelberg, Im Neuenheimer Feld 400, 69120 Heidelberg, Germany

**Keywords:** Autologous cranioplasty, CAD/CAM implant, Cranioplasty, Decompressive craniectomy, Multidrug-resistant bacteria, PMMA

## Abstract

Cranioplasty (CP) is a standard procedure in neurosurgical practice for patients after (decompressive) craniectomy. However, CP surgery is not standardized, is carried out in different ways, and is associated with considerable complication rates. Here, we report our experiences with the use of different CP materials and analyze long-term complications and implant survival rates. We retrospectively studied patients who underwent CP surgery at our institution between 2004 and 2014. Binary logistic regression analysis was performed in order to identify risk factors for the development of complications. Kaplan-Meier analysis was used to estimate implant survival rates. A total of 392 patients (182 females, 210 males) with a mean age of 48 years were included. These patients underwent a total of 508 CP surgeries. The overall complication rate of primary CP was 33.2%, due to bone resorption/loosening (14.6%) and graft infection (7.9%) with a mean implant survival of 120 ± 5 months. Binary logistic regression analysis showed that young age (< 30 years) (*p* = 0.026, OR 3.150), the presence of multidrug-resistant bacteria (*p* = 0.045, OR 2.273), and cerebrospinal fluid (CSF) shunt (*p* = 0.001, OR 3.137) were risk factors for postoperative complications. The use of titanium miniplates for CP fixation was associated with reduced complication rates and bone flap osteolysis as well as longer implant survival rates. The present study highlights the risk profile of CP surgery. Young age (< 30 years) and shunt-dependent hydrocephalus are associated with postoperative complications especially due to bone flap autolysis. Furthermore, a rigid CP fixation seems to play a crucial role in reducing complication rates.

## Introduction

Cranioplasty (CP) is a standard surgical procedure in patients after (decompressive) craniectomy. Nevertheless, a standardized way of performing CP has not yet been established, and the procedure is associated with complication rates of up to 36% [3, 5, 17, 20, 22, 27]. Recently, an increased interest in analyzing possible factors associated with complications has emerged in order to improve modalities of the procedure. Various potential risk factors have been identified: CP timing, optimal CP material (autologous vs. alloplastic), bone resorption rate using autologous CPs, or possible risk factors that may influence the implant survival [1, 2, 12–14, 16–18, 24]. Possible factors such as hydrocephalus, patient age (< 30 years), and segmented bone flaps may lead to significant higher rates of complications and bone flap resorption [24]. A combination of an autologous implant and a younger age seems to play an important role due to a high number of bone flap resorption. In cases of bone flap resorption in children and adolescents, subsequent revision is necessary in up to 50% of cases [6, 16, 23]. Furthermore, the use of alloplastic materials (PMMA) seems to have a lower revision rate, especially by avoiding bone autolysis [7]. However, the majority of reports have included limited patient numbers and did not analyze long-term results. Follow-up examinations of CP patients can be difficult due to the heterogeneity of craniectomy indications (e.g., traumatic brain injury, stroke, subarachnoid hemorrhage, or tumor). Nevertheless, long-term follow-up examinations are necessary due to the fact that complications like bone flap osteolysis often occurs after a considerable period of time.

The aim of this study was to identify risk factors in a large patient collective for the occurrence of complications after CP as well as factors that have an influence on implant survival. Specific focus was drawn to a long follow-up, covering delayed complications like bone flap osteolysis or implant loosing as well as the behavior of alloplastic implants during the long course.

## Methods

### Patient characteristics and study design

The present study was designed retrospective and consecutive. The study protocol was approved by the local ethics committee. In accordance with the ethical decision, patient consent was not required due to a purely retrospective analysis of existing data. No additional patient data collection or intervention was necessary. Furthermore, analysis was performed anonymized.

A total of 508 CPs in 392 patients have been performed at our university hospital between 2004 and 2014 and were included in this analysis. We included all patients who required decompressive craniectomy as well as patients who required craniectomy and bone flap replacement due to infections or tumors. There were no exclusion criteria. The medical records were retrospectively analyzed with specific focus on patient demographics, specific risk factors (e.g., nicotine or drug abuse, diabetes mellitus, multidrug-resistant bacteria), imaging results, and surgical treatment modalities. Documentation of multidrug-resistant bacteria (MDRB) includes a preoperative germ colonization (e.g., nose, skin, anal) before the initial CP without signs of florid inflammation (e.g., fever, elevated white blood cells, etc.). Furthermore, the in-hospital course and long-term outcome were analyzed. The length of follow-up was based on the available patient data.

### CP surgery

CP surgery was performed under general anesthesia. ﻿A perioperative antibiotic prophylaxis with cephazolin 2 g was administered 30 min prior to surgery. In case of penicillin allergy, patients received clindamycin 900 mg. The patient was usually positioned supine. After shaving and disinfecting the head, skin incision was reopened. The bony defect was prepared, if possible, without injuring the dura. After complete preparation of the defect, cranioplasty was inserted and fixed. In the case of an existing cryopreserved autologous bone flap, this was primarily used. If the autologous implant was missing (e.g., infection), an implant made of alloplastic material was used. Two major types of alloplastic implants were used: (1) hand-molded PMMA implants (Palacos®) supplemented with antibiotic agents (Gentamycin) or (2) premanufactured patient-specific implants, “Computer Aided Design and Manufacturing” (CAD/CAM) made of PMMA (Zimmer Biomet®), PEEK (Synthes CMF®), or titanium (Craniotomy Construct Bochum GmBH®). All CAD/CAM PMMA implants were placed in an antibiotic solution (Gentamycin, 80 mg, Ratiopharm) before implantation. The implant was fixed with sutures, bone clamps, or titanium plates depending on the surgeon’s preference. Afterwards, the skin was closed in a typical way. All CP surgeries were performed by a team of an experienced consultant neurosurgeon and a junior resident. A specialization of the surgeon (e.g., vascular or tumor) was not necessary. A standard postoperative antibiotic prophylaxis was not administered, only at the individual request of the surgeon.

### Follow-up

After a successful operation, the patients were usually hospitalized for 5 days. Cranial computer tomography (CCT) was not performed routinely after surgery and only in cases of clinical deterioration. The follow-up was carried out in our outpatient clinic, usually after 3 months and at 1- to 5-year intervals. Follow-up was independent of the underlying diagnosis. The patient was examined, and possible complications (e.g., wound healing disorders, bone flap autolysis) were identified. If the patient showed clinical signs of bone flap autolysis (e.g., new or increasing skin retractions due to osteolytic deformation or bone flap loosening), a CCT was performed in order to quantify autolysis. Routine CCT imaging was not performed. For the present study, bone flap osteolysis/necrosis was defined as a partial or complete resorption of the bone in CCT scans as well as presence of clinical signs. Indications for revision surgery due to bone flap autolysis were bone flap loosening, large osteolytic areas (> 4 cm), and increased risk of falling as well as patient discomfort. In cases of small osteolytic areas and/or thin bone in CCT images, a conservative treatment was performed with short-term follow-up examinations.

Bone flap autolysis revision surgery was performed in a single-step procedure with removal of the osteolytic bone and reinsertion of a new alloplastic implant. Two-step surgery with explantation of CP and secondary reinsertion after a time period of at least 3 months was performed in cases of subcutaneous infections and/or bone flap osteomyelitis. In the event of immediate complications (e.g., infections), the patient was admitted to the hospital. All follow-up examinations and inpatient hospital stays were documented and available for data analysis.

### Statistical analysis

Data were collected in an Excel database and were statistically analyzed with a standard SPSS software package (Version 25, IBM Corp.). Absolute and relative frequencies are presented as mean and standard deviation. A critical difference of 5% (*p* < 0.05) was assumed to be statistically significant. Binary univariate logistic regression analysis was used to determine which factors were associated with complications that needed surgical revision. Selection of test variables was based on the available literature and the personal experience of the authors. Survival rates of the primary implants were determined using Kaplan-Meier survival analysis. In order to identify factors that had an influence on the survival rates, multivariate Cox regression analysis was performed. Variable selection were based on background knowledge of the authors and a careful literature review. Depending on the number of patients, the maximum number of test variables (*n* = 16) for binary logistic regression as well as multivariate Cox regression was limited. Log-rank test and Cox regression analysis were used to compare the survival rates of the different materials used for the primary CP.

## Results

### Initial surgery (craniectomy)

A total of 392 patients were included in the analysis, of which 210 (53.3%) were male and 182 (46.7%) were female. The mean age at the time of craniectomy was 45.4 ± 15.9 years. The most common underlying pathologies requiring craniectomy were space-occupying cerebral infarction (30.6%; *n* = 120), traumatic brain injury (26.3%; *n* = 104), and aneurysmal subarachnoid hemorrhage (17.8%; *n* = 70) (Table 1). Furthermore, patients with craniectomy due to primary (e.g., encephalitis) or secondary infection (e.g., bone flap osteomyelitis after initial surgery) as well as bone destructive tumors (e.g., meningiomas) were included. The main patient-specific risk factors included arterial hypertension (45.1%; *n* = 177), nicotine abuse, (30.9%; *n* = 121), and the intake of oral anticoagulants (22.4%; *n* = 88) or platelet aggregation inhibitors (21.9%; *n* = 86). In the majority of cases (89.3%; *n* = 350), craniectomy was performed one sided, with a mean size of 122 × 84 mm (Table 1). The overall surgery-related complication rate of craniectomy (< 30 days) requiring surgical revision was 11.9% (47/392). The most common complications were early postoperative hemorrhages (6.9%; *n* = 27) and wound healing disorders (3%; *n* = 12).

**Table 1:** Details of patient population (*n* = 392) (*n* number; %, proportion)

Age		
Mean ± SD	45 ± 15.9 years	
Age categories	*n*	%
0–30 years	68	17.3
30–60 years	268	68.4
> 60 years	56	14.3
Gender	*n*	**%**
Male	210	53.3
Female	182	46.7
Decompressive craniectomy (mean size 128 × 86.6 mm)	*n*	**%**
Space-occupying cerebral infarction	120	30.6
Traumatic brain injury	104	26.5
Subarachnoid hemorrhage	70	17.8
Spontaneous intracerebral hemorrhage	33	8.4
Non-traumatic sub-/epidural hematoma	6	1.5
Venous sinus thrombosis	7	1.8
Craniectomy (mean size 82 × 66.6 mm)	*n*	**%**
Infection	34	8.7
Primary	7	1.8
Secondary	27	6.9
Tumor	18	4.6
Technique	*n*	**%**
Unilateral	350	89.3
Bilateral	7	1.8
Bifrontal	17	4.3
Tumor craniectomy	18	4.6

### Cranioplasty

All 392 patients underwent primary CP surgery after an average time of 158 days ± 240 after craniectomy. Of these patients, 103 were subjected to one or more revision CP surgery due to complications (*n* = 116), resulting in a total of 508 CP procedures. The mean follow-up of all CP patients was 91.5 ± 47.5 months. Differences were observed in the age groups: younger patients (< 30 years) were followed for a mean time of 87.25 ± 57.4 month, patients between 30 and 60 years for 64.54 ± 49.6 month, and older patients (> 60 years) for 44.93 ± 38.9 months. Patient-specific risk factors before primary CP are shown in Table 2.

**Table 2:** Patient-specific risk factors before primary CP (*n* number; %, proportion)

	*n*	(%)
Arterial hypertension	177	45.1
Diabetes mellitus	49	12.5
Other cardiovascular risk factors (e.g., coronary artery disease)	113	28.8
Current smoker	121	30.9
Drugs and/or alcohol abuse	49	12.5
Renal insufficiency	9	2.3
Liver cirrhosis	25	6.4
Coagulation disorders	19	4.8
Anticoagulant	88	22.4
Platelet aggregation inhibitors	86	21.9
Adiposities	53	13.5
Cachexia	3	0.7
Immunosuppression	5	1.3
Multidrug-resistant bacteria (MDRB)	56	14.3

In the context of primary CP, the majority of patients (39.8%; *n* = 156) underwent CP after 90 to 180 days following craniectomy, 28.1% (*n* = 110) after 30 to 90 days, and 20.9% (*n* = 82) after > 180 days (Table 3). Only 2.8% underwent ultra-early CP (< 30 days after craniectomy), and 5.1% of patients were subjected to a single-step surgery (e.g., in cases of simultaneous tumor resection and CP surgery). Different materials were used for reconstruction (Table 4). Autologous bone graft was used in most cases (57.3%; *n* = 291) followed by hand-molded PMMA implants (Palacos®, 19.1%; *n* = 97). In cases of autologous CP, 67 patients (23%) had a fragmented bone flap with two or more bone fragments. Patient-specific implants, “Computer Aided Design and Manufacturing” (CAD/CAM) made of PMMA, PEEK, or titan, were used in 115 cases (22.6%), especially for revision CP (62%; *n* = 72).

**Table 3:** Details of CP surgery (*n* number; %, proportion)

Time between DC and CP	*n*	%
Ultra early (< 30 days)	11	2.8
Early (30–90 days)	110	28.1
Late (90–180 days)	156	39.8
Prolonged (> 180 days)	82	20.9
Unknown	12	3.1
Simultaneous craniectomy + CP	21	5.4
Duration of CP surgery		
Primary CP	131 ± 48 min	
Revision CP	159 ± 65 min	
CSF shunt	*n*	**%**
Before primary CP	57	14.5
After primary CP	17	4.3
Simultaneous shunt + CP	15	3.8
During long-term follow-up	5	1.3

**Table 4:** Distribution of different materials for primary CP (*n* = 392) and revision CP (*n* = 116) (*n* number; %, proportion)

Material	Primary CP		Revision CP		Total	
	*n*	%	*n*	%	*n*	%
Autologous bone	289	73.7	2	1.7	291	57.3
PMMA manually (Palacos®)	57	14.5	40	34.5	97	19.1
PMMA CAD/CAM (Biomet®)	24	6.1	34	29.3	58	11.4
PEEK (CAD/CAM)	14	35.7	28	24.1	42	8.2
Titanium mesh	3	0.8	2	1.7	5	1
Titanium (CAD/CAM)	5	1.3	10	8.6	15	3

### Surgical details

Mean duration of surgery for primary CP was 131 ± 48 min and 159 ± 65 min for revision CP (Table 3). Implant fixation was performed with titanium osteosynthesis miniplates (42.3%; *n* = 215) or bone clamps (40.2%; *n* = 204, CranioFix®) in the majority of cases. Sutures only were used for fixation in 8.7% (*n* = 44), and all other cases received a combination of the mentioned materials.

In 109 of 508 CP surgeries, cerebrospinal fluid (CSF) reduction was necessary prior to or during surgery due to hydrocephalus or a bulging defect which had interfered with CP placement. In a total of 94 patients (24%), a CSF shunt was necessary due to hydrocephalus. Whereas 72 out of 392 patients (18.4%) had an indication for shunt placement before primary CP, only 4.3% of patients (17/392) received the shunt after primary CP and another 1.3% (5/392) during the long-term follow-up. The majority of patients with initial shunt indication underwent separate shunt surgery before CP (64%, *n* = 57). Simultaneous shunt insertion and CP were performed in only 15 patients (16.8%) (Table 3).

### Complication rates

All complications after primary and revision CP were analyzed. We regarded all those complications to be “main complications” that required CP revision or surgical intervention as well as surgery-related death. Overall complication rate was 32.9% (*n* = 167), divided in primary CP surgery 33.2% (*n* = 130) and 32.2% (*n* = 37) for revision surgery (Table 5). A total of 116 patients required a revision of CP due to complications requiring explantation of the primary CP. Of these 116 patients, 92 had two CPs, ten patients needed three CPs, and one patient needed a total of five CPs.

**Table 5:** Major complications resulting in surgical revision after CP (including explantation of cranioplasty)

	Primary CP		Revision CP	
	*n*	%	*n*	%
Wound healing disorders	15	3.8	11	9.5
Osteomyelitis/graft infection	31	7.9	8	6.9
Bone flap osteolysis	43	11.0	–	–
Loosening of CP	15	3.8	5	4.3
CSF leakage	6	1.5	1	0.8
Epidural/subdural hematoma	12	3.1	10	8.7
Intracerebral hematoma	1	0.2	–	–
Peri-/postoperative mortality	1	0.2	–	–
Total	130	33.2	37	32.2

The major complications after primary CP were osteolysis of the autologous bone graft (*n* = 43). In young patients (< 30 years), osteolysis occurred after an average time of 38 ± 35.8 months, while patients between 30 and 60 years had revision surgery after a mean time of 23.8 ± 19.8 months. Only one patient aged over 60 years suffered from osteolysis after 25 months. Other complications were infection of the graft material (*n* = 31) and wound healing disorder as well as loosening of the graft (*n* = 15 each). Main complications after revision CP were wound healing disorder (*n* = 11), followed by epi- or subdural hematoma (*n* = 10) and graft infection (*n* = 8). Overall, only one patient died during the hospital stay.

### Influencing factors

Binary logistic regression analysis was performed to determine which factors have an influence on complication rates (Table 6). A significant increase of complications was observed in young patients (0–30 years) at time of craniectomy (*p* = .026, OR 3.150), colonization with MDRB in the patient’s medical history (*p* = .045, OR 2.273), and the presence of a CSF shunt (*p* = .001, OR 3.137). The use of titanium miniplates for CP fixation had a significant positive impact on complication rates (*p* = .013, OR 0.310).

**Table 6:** Binary logistic regression analysis for complication rates after primary CP (*OR* odds ratio; *significant negative influence; ^#^significant positive influence)

	*p* value	OR	95% KI
Age			
0–30 years	0.026*	*3.150*	*1.143–8.639*
30–60 years	0.386	1.434	0.634–3.242
> 60 years	0.386	0.697	0.308–1.576
Patient-specific risk factors			
Nicotine	0.441	1.280	0.683–2.396
Diabetes	0.513	0.727	0.280–1.888
Fragmented autologous bone	0.275	1.487	0.730–3.029
Multidrug-resistant bacteria	0.045*	*2.273*	*1.018–5.074*
Time between CPs			
< 30 days	0.154	2.340	0.727–7.531
30–90 days	0.635	0.809	0.337–1.942
90–180 days	0.142	0.541	0.238–1.229
Others			
Surgery time for CP < 130 min	0.870	1.050	0.583–1.892
Alloplastic CP CAD/CAM	0.332	0.436	0.081–2.335
Alloplastic CP non CAD/CAM	0.114	4.333	0.703–26.719
Implant fixation with miniplates	*0.013*^#^	*0.310*	*0.123–0.781*
Implant fixation with clamps	0.336	1.611	0.609–4.259
CSF shunt	0.001*	*3.137*	*1.613–6.101*

Binary logistic regression analysis was also performed for the two most common CP fixation materials (titanium miniplate vs. bone clamp). In case of autologous CPs, titanium miniplates showed a significant lower rate of bone flap osteolysis (Co-eff − 1.414; *p* = .029) than bone clamps (Co-eff 0.5; *p* = .261).

### Implant survival

**Fig. 1:**
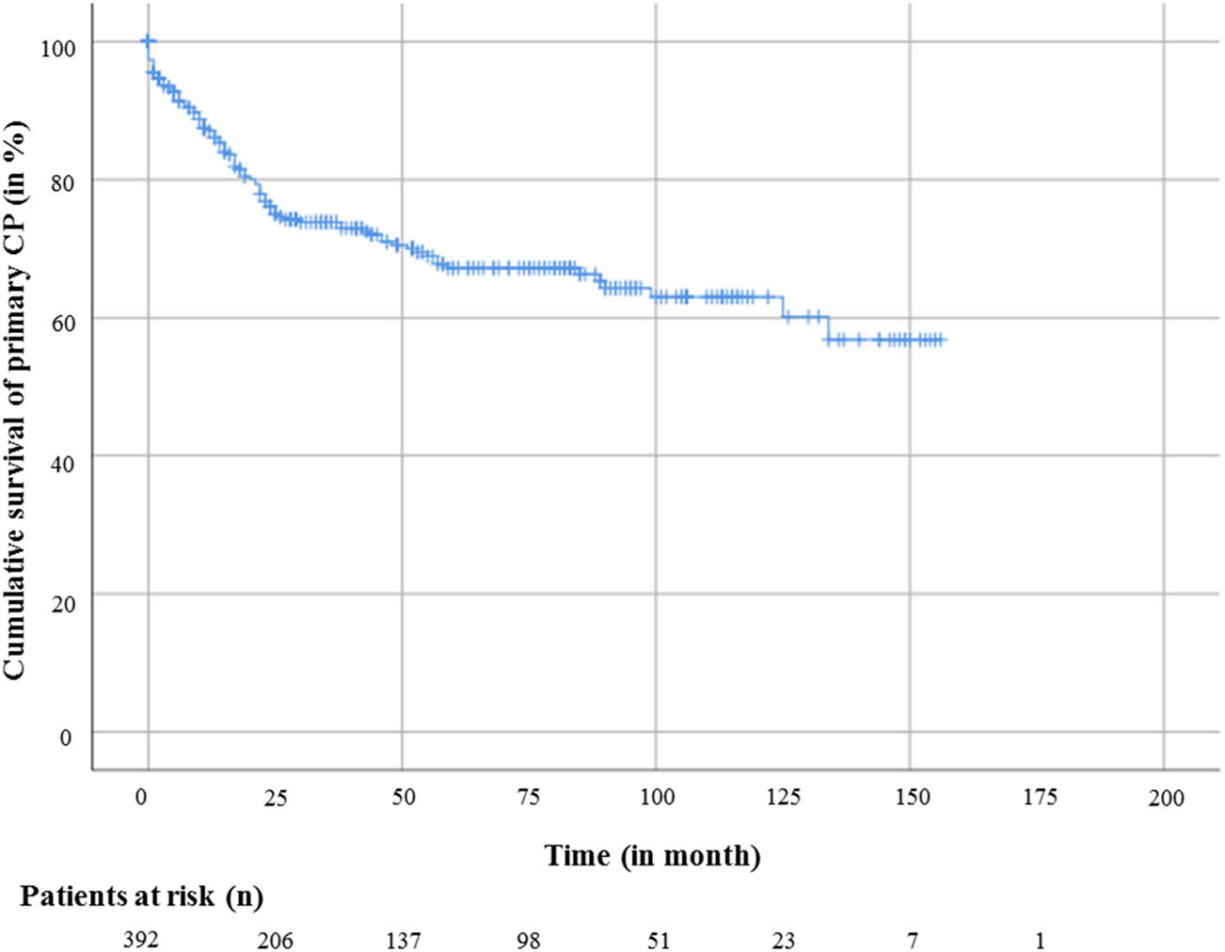
Kaplan-Meier estimator for analysis of implant survival of the primary CP with the number of patients at risk (*n*)

Kaplan-Meier analysis was used to determine the mean survival time for the first CP from implantation to explantation due to complications (Fig. 1). The estimated overall survival time of the primary CP was 120 ± 5 months. Multivariate Cox regression analysis was used to determine which factors had an influence on the implant survival time. Early reconstruction < 30 days after craniectomy (*p* = .006, HR: 3.008, CI: 1.371–6.602) and the presence of a CSF shunt (*p* = .040, HR: 1.696, CI: 1.023–2.812) were identified as factors with significantly negative influence on the survival rate of the primary CP. The use of titanium miniplates for CP fixation had a significantly beneficial influence on the survival rate of the first CP (*p* = .009, HR: 0.351, CI: 0.161–0.767). Further analysis included patient-specific risk factors (nicotine, diabetes, previous surgical intervention, MDRB), patient age, all time periods between DC and CP, as well as intraoperative usage of antibiotics, but all factors showed no statistically significant differences.

**Fig. 2:**
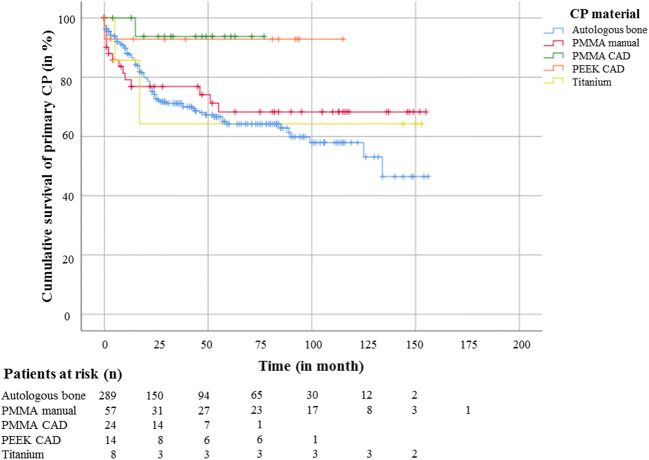
Kaplan-Meier estimator for analysis of implant survival of the primary CP with the number of patients at risk (*n*) classified for all CP materials

To compare the survival time of different CP materials after primary surgery, another Kaplan-Meier analysis was performed followed by a Log-rank test and Cox regression analysis (Fig. 2). Best survival rates were found for PMMA- and PEEK-CAD/CAM implants. Furthermore, implant survival analysis showed a significant advantage (*p* = .038) of the PMMA-CAD/CAM implant (HR: 0.170, CI: 0.024–1.218) compared with autologous bone grafts (HR: 1.69; CI: 1.043–2.833). The survival rates of all other materials showed no significant difference.

## Discussion

### Complication rates

Even though CP is a common procedure in neurosurgical practice, it is associated with considerable complication rates. In the present study, we found complication rates of approximately 33% in primary and revision surgery. These findings are well corresponding to the data reported in previous studies which have reported complication rates of up to 40% [5, 13, 14, 17, 26, 27]. A systematic review and meta-analysis by Malcolm et al. show a ﻿pooled overall complication rate of 19.5% (*n* = 609/3126) and surgical revision rate of 13.2% ﻿(*n* = 191/1445) across all studies [13]. Compared with the present work, the pooled complication rate of Malcolm et al. was lower, but the individual complications of both studies showed no significant differences. In detail, infection rate (7.7% vs. 7.9%) was similar in both studies; rate of intracranial hemorrhage (4.9% vs. 3.3%) was lower in the present study; and rate of bone flap autolysis and loosening (9.3% vs. 14.8%) was higher in the present study. These individual variations are attributable to the retrospective nature of single-center studies. Nevertheless, the long follow-up period of about 8 years in the present study represents a typical CP patient’s course.

### Influencing factors

Like other studies, we also analyzed risk factors which are familiar with higher complication rates. In the present study, a younger age (< 30 years), the colonization with MDRB, and a CSF shunt dependency were main risk factors which significantly increased the rate of postoperative complications after CP. The use of titanium miniplates for CP fixation had a beneficial impact on complication rates and overall implant survival time.

### Age

The impact of patient age and CSF shunt dependency has previously been identified as a risk factor for postoperative complications [7, 24]. Martin et al. have reported a surgical revision rate of 54.4% in pediatric patients due to bone flap osteolysis, which was associated with younger age and permanent shunt placement [16]. However, other authors have reported an increased complication rate after CP surgery in patients with an age > 60 years [3, 27]. The increased rates of complications and revision surgery, especially in younger patients, may be caused by the use of the autologous material [14]. Younger patients show significantly higher rates of bone flap osteolysis and are more dependent on the implant during their lifetime. Despite longer follow-up periods in younger patients (< 30 years), occurrence of osteolysis (38 ± 35.8 vs. 23.8 ± 19.8 months) differed only about 1 year compared with the main age groups (< 30 years vs. 30–60 years). Therefore, the higher osteolysis rate in younger patients cannot be explained by longer follow-up periods. The pathophysiological mechanism of bone flap osteolysis remains unclear. There is a suspicion in young patients that osteolysis is caused by an imbalance of internal bone resorption and external apposition [16]. In contrast, older patients may show less osteolysis reaction but have an increased overall risk profile due to underlying comorbidities which can cause higher rates of wound healing disorders, infections, and postoperative hemorrhage.

### CSF shunt

As previously described, the CSF shunt is an independent risk factor for increased overall complication rates and reduced implant survival time. This is evident both in our work and in the literature [8, 16, 18, 24, 28]. The recent analysis showed no significant difference in complications for the timing of CSF shunt surgery (staged vs. simultaneously) in our study. ﻿Nevertheless, Mustroph et al. could show in a large ﻿systematic review and meta-analysis that simultaneous procedures were associated with increased complication rates compared with staged procedures [18]. The lack of significance in our study can be explained by the small number of simultaneous interventions.

Nevertheless, patients with CP and CSF shunt should be informed about the higher risk profile of CP surgery. Furthermore, the postoperative course and follow-up visits should include a focus on over-/underdrainage of the CSF shunt and bone flap osteolysis/loosening/sinking in autologous CP.

### MDRB

The impact of MDRB on complication rates seems plausible as colonization with these microorganisms has to be considered a co-marker of severe morbidity. Although data on neurosurgical interventions are not available, neurological and geriatric patients have a higher risk (up to 22.7%) of MDRB colonization [9]. Furthermore, MDRBs are associated with overall higher hospital costs, significantly longer intensive care unit and hospital stay, as well as increased morbidity and mortality in orthopedic and cardiologic patients [19, 25].

### CP fixation

The use of the CP fixation material seems to have an impact on postoperative complication rates. We demonstrate that titanium miniplates significantly reduced the risk of overall postoperative complications as well as bone flap osteolysis compared with the use of autologous implants. Furthermore, overall implant survival time was significantly longer. Other studies reported similar results with an advantage of titanium plates [3, 21]. In addition, a recent study ﻿demonstrated a higher rate of successful bone fusion in cases in which plates and screws were used [10]. Furthermore, the authors showed that bone resorption was considered to occur in a solitary bone flap without bone fusion or in portions of the bone flap far away from the fusion site. These findings underline our assumption that a superior bone fusion is associated with a lower rate of bone flap autolysis and loosening. Therefore, the use of miniplates for CP fixation seems to be associated with a lower risk of bone flap autolysis.

### CP timing

Further risk factors for CP complications include timing of CP (early vs. late) and the type of implant material [13–15, 17]. In this study, we did not find any significant relation between certain timeframes and increased complication rates. Nevertheless, further regression analysis showed that an early CP (< 30 days after craniectomy) was an independent risk factor for a decreased implant survival. Previous studies have reported that early CP surgery (< 90 days) is associated with an increase odd of hydrocephalus and﻿ highest risk of infection within 14 days of initial craniectomy, whereas CP between 15 and 30 days ﻿minimizes risk of infection, seizure, and autologous flap resorption [13, 17].

### Implant material and survival

Regarding the implant material, we did not find any correlation between the use of alloplastic materials and increased infection rates. Nevertheless, implant survival time of PMMA-CAD/CAM implants was significantly longer compared with autologous bone grafts. Bobinski et al. and Kim et al. showed similar results with a longer survival time of alloplastic implants and reduced reoperation rates [2, 11]. The longer survival of alloplastic CP can be explained by the absence of bone flap resorption, which is the main risk factor of autologous implants in up to 20% of cases [11]. Therefore, alloplastic CPs may reduce the rate of revision surgery and complication especially in patients < 30 years. Nevertheless, CP material for pediatric patients should be discussed very carefully due to the ongoing growth of the skull [7].

### Strengths and limitations of the study

The particular strength of the present study is the large patient population and the possibility of direct comparison of various influencing factors and surgical techniques for a very long follow-up period. All patients were routinely followed up for a long time independent of their underlying diagnosis. However, the study has several important limitations. The study was a retrospective work, reducing the level of evidence because of the possible presence of uncontrolled confounding factors as well as missing or biased data. Further studies (preferably RCTs) are necessary to prospectively analyze the modalities of autologous and alloplastic CP surgery with regard to complication rates and possible influencing factors as well as neurological outcome [4].

## Conclusion

The results of this single-center analysis demonstrate that CP surgery is associated with considerable complication rates of up to 33%. In the present study, young age (< 30 years), the presence of MDRB, and CSF shunt dependency were risk factors for postoperative complications after CP. Furthermore, young age, CSF shunt dependency, and early CP (< 30 days after craniectomy) are risk factors for a reduced overall implant survival time. A positive influence could be shown for the titanium miniplate fixation system. The use of titanium miniplates for CP fixation was associated with reduced rates of postoperative complications and longer implant survival times. Longer implant survival times were observed in patients treated with PMMA-CAD/CAM implants compared with autologous bone. Nevertheless, further studies are necessary to prospectively analyze the modalities of autologous and alloplastic CP surgery.
